# Experimental Evaluation of Sheep Wool Fibres as Sustainable Reinforcement in Eco-Friendly Cement Mortars

**DOI:** 10.3390/ma19020427

**Published:** 2026-01-22

**Authors:** Carlos Ruiz-Díaz, Guillermo Guerrero-Vacas, Óscar Rodríguez-Alabanda, Manuel Cabrera, Julia Rosales

**Affiliations:** Department of Mechanical Engineering, University of Córdoba, 14071 Córdoba, Spain; crdiaz@uco.es (C.R.-D.); guillermo.guerrero@uco.es (G.G.-V.); orodriguez@uco.es (Ó.R.-A.); manuel.cabrera@uco.es (M.C.)

**Keywords:** sheep wool fibres, cementitious mortars, natural fibre reinforcement, flexural strength, compressive strength, thermal conductivity, eco-efficient materials, sustainable construction, circular economy

## Abstract

Sheep wool is a low-value agricultural by-product with potential to contribute to more sustainable cementitious materials. This study investigates Segureña sheep wool fibres as reinforcement in cement mortars, comparing washed wool (W) and cement-encapsulated wool (E) at the same oven-dry raw wool dosages (0.5, 1.0, and 3.0 g per batch), and benchmarking against polypropylene (PP) fibres. Flexural and compressive strength were evaluated at 1, 7, and 28 days, whereas apparent density, water absorption, and thermal conductivity were assessed at 28 days. An intermediate dosage (1.0 g per batch) provided the most favourable mechanical response, while the highest dosage (3.0 g per batch) reduced performance, plausibly due to dispersion limitations and void formation. At 28 days, W-1 reached 9.65 ± 0.50 MPa in flexure (very close to PP-1) and 59.70 ± 1.05 MPa in compression, exceeding PP-1 in compression. Wool incorporation also reduced apparent density and yielded an observed reduction in thermal conductivity of up to ~18% at the highest dosage (single specimen per series). Overall, optimally dosed washed wool can deliver competitive mechanical performance while improving thermal behaviour, supporting circular-economy valorisation of waste wool in eco-mortars.

## 1. Introduction

The construction sector remains one of the main contributors to global environmental impact, accounting for a considerable share of energy use, greenhouse-gas emissions, and non-recyclable waste. It is estimated to be responsible for approximately 5–8% of global anthropogenic CO_2_ emissions, when both process- and energy-related emissions from cement are considered [1,2]. In view of these challenges, there has been a clear shift towards eco-efficient solutions, in which renewable or waste-derived components within cementitious systems help to advance circular-economy objectives [3].

In parallel with these developments, the accumulation of low-grade sheep wool driven by the rise in synthetic fibres has generated environmental and economic burdens in producer regions [4]. Nevertheless, the biological origin and biodegradability of wool make it a promising candidate for valorisation within building materials.

Sheep wool is a keratin-based fibre with a hierarchical cuticle–cortex structure, which confers elasticity, resilience, and moisture-buffering capacity [5]. These attributes underpin its thermal and acoustic insulation performance, as well as its intrinsic fire retardance associated with nitrogen content and bound water [6]. In building applications, sheep wool typically exhibits thermal conductivity within the 0.034–0.067 W·m^−1^·K^−1^ range, comparable to common insulators while offering low embodied energy and favourable end-of-life options [4]. Independent experimental studies likewise report values close to 0.032–0.048 W·m^−1^·K^−1^ for wool-based insulation products [7].

Within cement and concrete technology, natural fibres (flax, hemp, sisal, coir, etc.) have been widely investigated to enhance toughness, crack-bridging capacity, and post-cracking behaviour of brittle matrices, although with trade-offs in fresh-state workability and durability that depend on fibre type and dosage [8]. In contrast, the use of animal fibres, particularly sheep wool, in cementitious composites is less explored, although it is gaining traction [9]. The available evidence indicates that wool fibres can increase fracture toughness and reduce brittleness, thereby offering eco-efficient alternatives to synthetic fibres [5]. A key challenge, however, is that the alkaline pore solution of Portland cement may degrade keratin and weaken fibre–matrix adhesion, which motivates the study of surface treatments or protective strategies. Such degradation has been reported to depend on the alkalinity of the cementitious matrix and exposure time, and it can be accelerated under sustained wet conditions (e.g., long-term curing in water), potentially affecting fibre effectiveness and long-term mechanical performance; therefore, protective strategies such as fibre encapsulation are of interest [10,11,12].

Beyond the mechanical response, the addition of wool fibres can reduce density and thermal conductivity by introducing a fibrous network and air voids, thereby enabling lightweight, insulating eco-mortars when dispersion, fibre length, and dosage are properly controlled [13]. Still, moisture sensitivity and processing effects typical of bio-based reinforcements must be addressed to ensure consistent performance [14].

Despite these promising indications, inconsistencies remain regarding the optimal fibre content, treatment, and geometry. Studies on rendering mortars report that high wool dosages and longer fibres can reduce compressive strength, despite improvements in cracking resistance [9]. Conversely, investigations on ultralight foamed systems identify approximately 1% wool by volume, combined with tailored fibre lengths and alkaline treatments, as a promising window, particularly with gains in flexural response [15]. These divergences underline the need for systematic, comparative evaluations under controlled procedures.

Although wool-reinforced mortars have been reported, existing studies often differ in fibre conditioning and mix-design choices, which complicates direct comparison and limits the isolation of treatment-driven effects [9,10,12,16,17]. The present study addresses this gap by (i) comparing washed wool and cement-encapsulated wool at the same oven-dry raw wool dosage, (ii) benchmarking against a polypropylene-fibre reference at matched dosages, and (iii) using a factorial/statistical approach to quantify the relative influence of dosage and treatment under controlled procedures.

In this context, the present work examines the feasibility of incorporating sheep wool fibres into cement mortars as a sustainable reinforcement and a potential lightweighting agent. Two fibre conditions are considered: (i) washed sheep wool fibres and (ii) the same washed fibres subsequently washed and encapsulated within a thin cementitious layer prior to mixing. This experimental design isolates the effect of the encapsulation barrier on fibre–matrix compatibility and on transport-related properties, while keeping the fibre origin constant. The mortars are evaluated in terms of mechanical (flexural and compressive), physical (density and absorption), and thermal performance, and are benchmarked against conventional mixtures under controlled procedures. In this way, the study clarifies the effects of fibre treatment and dosage and contributes to the development of new eco-concrete formulations aligned with circular-economy valorisation of agricultural by-products.

## 2. Materials and Methods

In this study, an experimental programme was developed to evaluate the influence of sheep wool fibres on the behaviour of lightweight mortar. The methodology included the preparation of fibres under two conditioning treatments, the production of mortar with different fibre incorporations, and the assessment of fresh, mechanical, thermal, and microstructural properties. The procedures described in this section were designed to ensure repeatability and comparability between mixtures and to allow a rigorous assessment of the effect of the fibres on the final composite.

### 2.1. Materials

#### 2.1.1. Cement

A commercial Portland cement (OPC) of strength class CEM I 42.5 R [18] (Cementos Portland Valderrivas, S.A, Alcalá de Guadaíra, Sevilla, Spain) was employed as the binder. This cement type is commonly used in studies involving mechanical testing of mortars due to its well-established performance and compliance with European specifications. The material was used as received, without any additional processing. The main composition and density of the OPC used is shown in Table 1.

#### 2.1.2. Aggregate

The fine aggregate consisted of a standard siliceous sand (CEN according to DIN EN 196) that met the gradation requirements typically prescribed for laboratory mortar testing. Its controlled particle-size distribution ensures adequate reproducibility and minimises the influence of aggregate variability on the results.

#### 2.1.3. Sheep Wool Fibres

**Figure 1:**
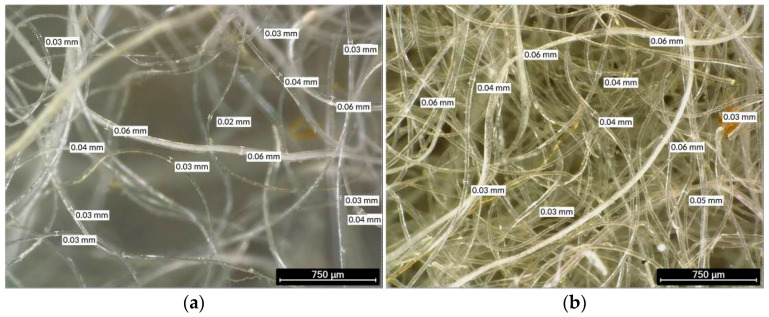
Measurements, appearance, and distribution of Segureña sheep wool reinforcement fibres obtained by optical microscopy: (**a**) Light white fibres with low straw residues; and (**b**) white/yellow fibres with low straw residues (different field of view).

Natural sheep wool fibres were incorporated as an organic addition to the mortar. The fibres originated from Segureña sheep, a traditional breed from the south-eastern Iberian region, and were supplied in their raw, untreated state by Wool Dreamers (Mota del Cuervo, Cuenca, Spain). Prior to their use, the fibres were conditioned according to two procedures, washing and cement encapsulation, intended to modify their interaction with the cementitious matrix and to enhance their stability in the alkaline environment. A representative microscopic characterisation of the fibres is provided in Figure 1, and Table 2 complements these qualitative observations with morphological and visual characterisation, including colour, type, and presence of residues, along with the maximum, minimum, and average diameters measured in the samples.

Figure 1a,b shows the heterogeneity of the fibres in thickness and arrangement, with diameters displaying significant variation and surface irregularities typical of unprocessed natural materials. Adapted from Ref. [21] (CC BY 4.0); edited for clarity.

#### 2.1.4. Water

Potable tap water was used for both the fibre treatments and the preparation of the mortar mixtures. The water quality is consistent with that generally accepted for cement-based composites.

### 2.2. Methods

The experimental procedures comprised the conditioning of the wool fibres, the preparation of the mortar mixtures, the fabrication of the specimens, and the subsequent curing regime. All steps were designed to ensure reproducibility and to allow a controlled assessment of the influence of the wool on the behaviour of the mortar. The methodology adopted follows established practices in the study of fibre-modified cementitious materials, adapted to the specific characteristics of the fibres and the objectives of the research.

#### 2.2.1. Wool Treatment Procedures

Two conditioning methods were applied to the wool fibres prior to their incorporation into the mortar: washing and cement encapsulation. These procedures were introduced to eliminate superficial impurities, promote more uniform dispersion, and enhance the fibres’ compatibility with the cementitious matrix. No cutting or sieving was applied, since cutting crimped/entangled wool is difficult to standardise and typically produces heterogeneous lengths. Therefore, fibres were used as received, and fibre length distribution was not quantified in this study.

1. Washed wool (W): Raw Segureña wool was manually teased and treated using water only (no detergents). Approximately 25 g of wool was placed in a container, and 300 g of water was added gradually to promote progressive wetting until saturation was achieved. The wetted fibres were then left to dry at ambient conditions for several days until no visible moisture remained. In this study, the term washed refers to this water-only soaking/conditioning followed by drying; no rinse cycles were applied. The dried fibres were manually separated prior to dosing in the mortar mixtures.
2. Cement-encapsulated wool (E): Encapsulated fibres were produced using a dilute cement slurry prepared with approximately 30 g of OPC and 600 g of water. The slurry was mixed until homogeneous and then poured over 25 g of teased wool, ensuring full immersion. The fibres were kept in contact with the slurry for approximately 3 days. When cement setting was not achieved under ambient conditions, the encapsulated fibres were oven-dried at 60 °C for 24 h, using a drying and sterilisation oven (Digitheat-TFT, J.P. Selecta S.A.U., Barcelona, Spain). After drying, the coated fibres were manually separated prior to incorporation into the mortar.

#### 2.2.2. Mortar Preparation

The mortar mixtures were prepared using controlled and reproducible proportions of cement, standardised sand, water, and fibres. A plain unreinforced control mixture (Control, 0 g fibres) was produced following the base mortar proportions prescribed in UNE-EN 196-1 [22] (450 g cement, 1350 g standard sand, and 225 g water). In addition to the sheep-wool-reinforced mixtures (washed and cement-encapsulated), polypropylene (PP) fibre-reinforced mortars were prepared as a synthetic reference using a commercial polypropylene microfibre product (SikaCim^®^ Fibras. Sika S.A. Spain, Alcobenda, Madrid, Spain), consisting of 6 mm monofilament polypropylene fibres for mortars and concretes. The product is CE-marked according to EN 14889-2 [23] (Class Ia, monofilament microfibres) and is manufactured by Sika S.A.U. (Alcobendas, Madrid, Spain).

All batches were prepared with constant cement, sand, and water contents, while the fibre mass was varied according to the experimental design. In all wool mixes, the stated fibre dosage (0.5, 1.0, and 3.0 g per batch) refers to the oven-dry mass of raw wool, and the same definition was used for both W and E to compare the effect of treatment at the same wool content. For E, the cementitious coating is an intrinsic outcome of the encapsulation treatment; coating pickup (coating mass fraction and variability) was not quantified in this study. Polypropylene reference mixes (PP-0.5, PP-1, and PP-3) were produced using the same fibre masses per batch adopted for wool (0.5, 1.0, and 3.0 g per batch) to enable direct comparison of dosage effects across fibre types. Fibre dosage is additionally reported as wt.% by mass of cement (bfoc), where 0.5, 1.0, and 3.0 g per batch correspond to 0.11, 0.22, and 0.67 wt.% bfoc, respectively.

Before mixing, each component was weighed individually using a precision balance (GRAM FS, Flintec S.L., Cardiff, UK) to ensure accurate dosing. The preparation procedure followed a standardised sequence. First, cement and water were introduced into the laboratory mixer (SJL–15L, Serve Real Instruments Co., Wuxi, China) and blended at low speed to initiate homogenisation. Sand was then added gradually, and the mixture was subsequently mixed at high speed to achieve full incorporation of the solid particles. When applicable, fibres (washed wool, cement-encapsulated wool, or polypropylene fibres) were added during the intermediate stage of mixing, allowing sufficient time for their dispersion while preventing agglomeration. This procedure ensured a uniform fibre distribution and consistent workability across mixtures.

All batches were mixed under comparable environmental conditions to minimise variability linked to temperature or humidity. The process yielded fresh mortar of adequate consistency for specimen fabrication.

Table 3 summarises the material dosages employed for each mixture, including the unreinforced control and the three fibre contents considered for washed wool, cement-encapsulated wool, and polypropylene fibres.

Due to specimen availability, density, water absorption, and thermal conductivity were obtained from single measurements (*n* = 1) per mix in this study; results are, therefore, discussed as observed trends rather than statistically significant differences. These properties are mainly governed by mixture composition and pore structure, and thermal conductivity is strongly related to density/porosity in cement-based materials [24,25,26].

#### 2.2.3. Moulding and Curing of Specimens

Two types of specimens were produced: the first type for evaluating physical and mechanical properties, and the second type for evaluating thermal properties.

1. 1. Prismatic Specimens

Prismatic specimens were prepared for the mechanical and physical characterisation of the mortar, including flexural and compressive strength (*f_u_*_,*f*_, *f_u_*_,*c*_), density, and water absorption. The fresh mortar was placed into standard steel moulds with internal dimensions of 40 × 40 × 160 mm. The moulds were filled in two layers, each compacted to eliminate entrapped air and to ensure a uniform density throughout the specimen. When required, compaction was performed using a horizontal compactor (111-100017/1, S.A.E. Ibertest, Madrid, Spain) to promote consistency across batches. The top surface was levelled to obtain a smooth finish, allowing accurate dimensional measurements and reliable test conditions.

2. Thermal Test Plates

For the thermal conductivity assessment, sample specimens were produced using rigid square moulds with internal dimensions of 300 × 300 × 30 mm. The fresh mortar was poured into the mould in a single layer and spread manually to ensure complete coverage of the base. The material was then levelled with a straight edge to obtain a uniform thickness, as variations in height could affect the steady-state heat transfer during the test. Light tapping of the mould was applied to minimise entrapped air, while avoiding intense mechanical compaction that could induce segregation in such a thin element. The finished surface was left plain and regular to ensure good contact with the measuring apparatus.

3. Curing Condition

After casting, all specimens, both prismatic and plate, were kept under controlled laboratory conditions for the first 24 h to allow initial setting. Once demoulded, they were stored in a curing chamber at (20 ± 1) °C and relative humidity ≥ 90% until testing. The specimens remained in this environment until the scheduled testing ages, ensuring a stable hydration process and minimising variability due to environmental fluctuations. This curing regime was applied uniformly to all mixtures, irrespective of fibre type or dosage.

### 2.3. Experimental Tests

A comprehensive testing programme was conducted to evaluate the behaviour of the mortar mixtures in both fresh and hardened states. The procedures described below were selected to provide a consistent and rigorous characterisation of workability, mechanical performance, thermal behaviour, and microstructural features. All tests were carried out under controlled laboratory conditions to ensure reproducibility and comparability across mixtures.

To provide a clear overview of the experimental design, Table 4 summarises the test programme, including the mixtures involved, the curing ages considered, and the type and geometry of the specimens used in each case. The Table also specifies the number of specimens tested for each procedure, reflecting the distinct specimen formats employed in the study, namely prismatic specimens for mechanical, density, and absorption tests, and plate specimens for thermal conductivity measurements.

The specific procedures for each test are described in the following subsections.

#### 2.3.1. Consistency Tests

The workability of the fresh mortar was assessed using a manual flow table apparatus and a standard truncated-cone mould, following UNE-EN 1015-3 [27]. The mortar was placed into a conical mould positioned at the centre of the table and compacted to eliminate entrapped air. The mould was then removed, after which the table was dropped a prescribed number of times, allowing the mortar to spread freely. The final spread diameter was measured along two perpendicular axes, and the average value was taken as the consistency of the mixture. This procedure enabled the evaluation of the influence of wool fibres on the fresh behaviour of the mortar.

#### 2.3.2. Mechanical Tests

Mechanical characterisation of the hardened mortar was performed through flexural and compressive strength tests according to UNE-EN 196-1 [22], using a hydraulic universal testing machine (IBMU4, S.A.E. Ibertest, Madrid, Spain) equipped with the corresponding flexural and compression fixtures and the prismatic specimens produced earlier.

Flexural strength was determined using a three-point bending configuration. Each specimen was placed on two supporting rollers and loaded at mid-span at a controlled rate until failure. The maximum load sustained was recorded, and the flexural strength was calculated based on the specimen geometry and loading conditions. This test provided an initial assessment of the mechanical response of each mixture.

The two halves resulting from the flexural test were subsequently used for compressive strength determination. Each half prism was positioned between steel plates and subjected to increasing axial load until failure. The compressive strength was calculated as the ratio between the maximum load and the cross-sectional area of the specimen. This test is widely used to evaluate cement-based materials and enables a quantitative comparison of the influence of fibre type and dosage on the mechanical properties.

#### 2.3.3. Physical Properties

Physical properties (apparent density and water absorption) were determined at 28 days following the experimental programme summarised in Table 4.

Specimens were oven-dried in a ventilated oven at 60 °C for 24 h and weighed to obtain the oven-dry mass, $m_{d}$. Apparent density was calculated as $\rho =m_{d}/V$, where V is the nominal specimen volume. The same oven-dry mass was used as the reference mass for water absorption.

For absorption, dried specimens were immersed in boiling water for 24 h to minimise air entrapment within the pore network; specimens were then removed, surface-dried with a cloth, and weighed to obtain the saturated mass, $m_{s}$. Water absorption was computed by Equation (1):

(1) $$WA\left(\%\right)=\frac{m_{s}-m_{d}}{m_{d}}\times 100$$

#### 2.3.4. Thermal Conductivity Test

The thermal conductivity of the mortar was measured by steady-state heat flux methodology, using a heat flow meter (FOX 600, TA Instruments, New Castle, DE, USA). Plate specimens were placed between a controlled heat source and a cold surface, creating a stable thermal gradient across the material. A heat flux sensor recorded the rate of heat transfer, while thermocouples monitored the temperatures at both interfaces. Thermal conductivity was calculated from Fourier’s law, based on the measured heat flux, the temperature difference, and the specimen thickness. This test allowed the assessment of how wool fibres influence the thermal insulating capacity of the mortar.

Prior to testing, plate specimens were conditioned in a ventilated oven at 60 °C for 24 h to minimise moisture-related variability, and then they were allowed to cool to the test temperature before measurement. Thermal conductivity was measured only for the control, W-3, and E-3 at 28 days, focusing on the highest wool dosage (3 g/batch) due to the time and specimen demands of plate testing [16,17,28].

#### 2.3.5. Microstructural Analysis

The microstructural features of the hardened mortar were examined using a digital microscope (Leica DVM6A, Leica Microsistemas S.L.U., L’Hospitalet de Llobregat, Spain). Small sample fragments were prepared and analysed to study the internal distribution of the fibres, their interaction with the cementitious matrix, and the presence of voids or discontinuities. Particular attention was given to the fibre–matrix interface, as well as to any signs of degradation or detachment. These observations complemented the mechanical and thermal results, providing qualitative insight into the composite behaviour.

### 2.4. Statistical Analysis

Statistical analysis was performed to quantitatively evaluate the influence of fibre treatment (washed or cement-encapsulated) and fibre content (0.5, 1.0, and 3.0 g per batch) on the mechanical performance of the mortars. Given the limited number of replicates and the factorial nature of the experimental design, the analysis was restricted to the 28-day mechanical responses, namely ultimate flexural strength (*f_u_*_,*f*_) and ultimate compressive strength (*f_u_*_,*c*_), which are the most representative indicators of hardened-state performance, and statistical outcomes are interpreted cautiously and primarily used to support observed trends.

General linear models (GLMs) were employed to assess the main effects of the studied factors and their interaction. Analysis of variance (ANOVA) was used to identify statistically significant effects at a 95% confidence level. Effect coding was adopted for categorical variables to allow an unbiased estimation of factor effects relative to the overall mean response. The fitted models were evaluated in terms of goodness-of-fit using the coefficient of determination (R^2^) and the standard error of the regression (S).

In addition, main effects plots based on adjusted means were generated to provide a graphical representation of the influence of fibre treatment and fibre content on the analysed responses. All statistical analyses were carried out using Minitab^®^ Statistical Software, version 19.0 (Minitab, LLC, State College, PA, USA).

## 3. Results and Discussion

The results obtained in this study are presented and discussed in this section, with emphasis on the properties governing the performance of hardened mortars reinforced with sheep wool fibres. The discussion is structured to analyse the influence of fibre treatment (washed and cement-encapsulated) and fibre dosage on the mechanical, physical, thermal, and microstructural behaviour of the composite material.

As part of the experimental programme, the fresh-state consistency of all mortar mixtures was evaluated by means of a flow table test. The results of this test indicated that, despite the incorporation of wool fibres, all mixtures exhibited adequate workability, allowing proper casting and specimen preparation without the need to modify the water-to-cement ratio or to use chemical admixtures. No practical limitations related to mixing, placement, or mould filling were observed during specimen fabrication.

Given the qualitative and control-oriented nature of this assessment, the flow table test was primarily used to verify the feasibility of incorporating wool fibres into the mortar matrix. Consequently, the discussion presented herein is focused on the properties of the hardened material that are directly related to structural and functional performance. The experimental results are interpreted by combining mechanical testing, physical characterisation, microstructural observations, and statistical analysis, in order to assess the suitability of sheep wool fibres as a sustainable alternative reinforcement in cementitious mortars.

### 3.1. Mechanical Performance

#### 3.1.1. Flexural Strength

The flexural strength results of mortars reinforced with sheep wool fibres at curing ages of 1, 7, and 28 days are presented in Table 5. The analysed mixtures include mortars reinforced with washed wool (W) and encapsulated wool (E), each produced with fibre contents of 0.5, 1, and 3 g per batch.

At early curing age (1 day), flexural strength values were relatively low for all wool-reinforced mortars, particularly those containing washed wool fibres. This behaviour can be attributed to the limited development of the cementitious matrix at this stage, combined with the presence of natural fibres, which may initially disturb matrix continuity and reduce early-age load-bearing capacity. Similar early-age trends have been reported in studies where natural fibres showed limited contribution to flexural resistance until the matrix had attained sufficient strength [29]. In contrast, the control mortars reinforced with synthetic fibres exhibited noticeably higher flexural strength values at 1 day, indicating a more effective crack-bridging action immediately after setting.

After 7 days of curing, a significant increase in flexural strength was observed for all mixtures. Washed wool-reinforced mortars showed a marked improvement compared to their early-age performance, reaching values between approximately 5.5 and 8.2 MPa, depending on fibre dosage. Mortars incorporating encapsulated wool generally exhibited slightly higher flexural strength values than those with washed wool, particularly for fibre contents of 0.5 and 1 g. This suggests that the encapsulation treatment enhances the fibre–matrix interaction, leading to a more efficient transfer of tensile stresses under bending loads, an effect also documented for treated natural fibres in cementitious composites [10].

At 28 days, flexural strength values further increased and tended to stabilise for all fibre-reinforced mortars. For both washed and encapsulated wool, the highest flexural strength was consistently obtained for mixtures with a fibre content of 1 g. Increasing the fibre dosage to 3 g did not lead to additional improvements and, in some cases, resulted in a slight reduction in flexural strength. Similar observations have been attributed to fibre clustering and increased void content at higher natural fibre contents, which locally weaken the matrix and limit effective strength transfer under flexural loading [9,30]. This non-linear effect of fibre dosage underscores the importance of optimising the fibre content in natural fibre-reinforced mortars.

**Figure 2:**
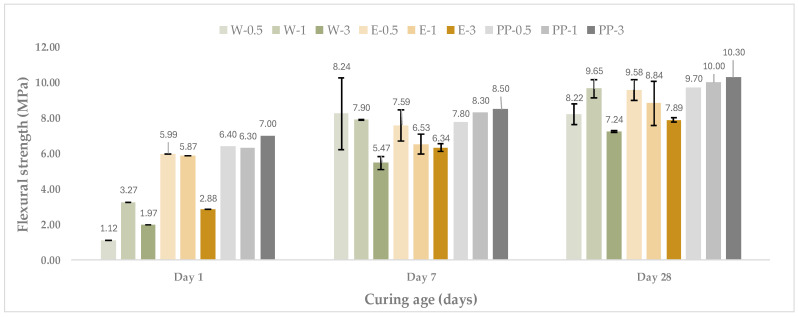
Evolution of flexural strength of mortars reinforced with washed and encapsulated sheep wool fibres at different curing ages, compared with a synthetic fibre reference. Error bars represent ±1 SD (n = 2 at 7 and 28 days; n = 1 at 1 day).

For comparison purposes, a control mortar reinforced with synthetic fibres (PP) was also tested under the same conditions, as shown in Figure 2.

When compared with the control mortar reinforced with synthetic fibres, wool-reinforced mortars exhibited lower absolute flexural strength values at all curing ages. However, the difference between wool-based and synthetic fibre mortars decreased with curing time, particularly at 28 days, where several wool-reinforced mixtures approached the flexural performance of the control system. This trend highlights the potential of sheep wool fibres as a viable natural alternative to conventional synthetic fibres, particularly when an appropriate fibre treatment and dosage are employed, as supported by recent reviews on wool reinforcement in cement-based materials [10].

#### 3.1.2. Compressive Strength

The compressive strength results of the wool-reinforced mortars at curing ages of 1, 7, and 28 days are presented in Table 6. As in the flexural analysis, the mixtures include mortars reinforced with washed wool (W) and encapsulated wool (E), with fibre contents of 0.5, 1, and 3 g per batch.

**Figure 3:**
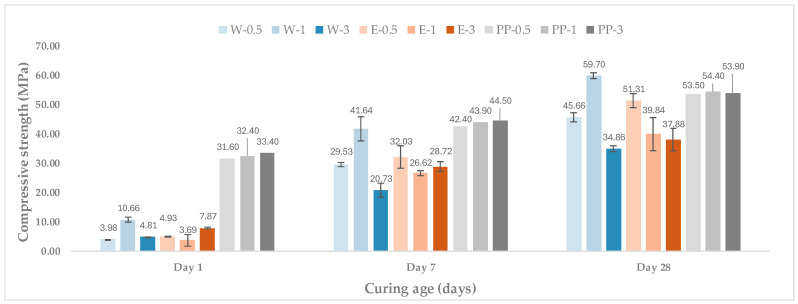
Evolution of compressive strength of mortars reinforced with washed and encapsulated sheep wool fibres at different curing ages, compared with a synthetic fibre reference. Error bars represent ±1 SD (n = 2 at 1 day; n = 4 at 7 and 28 days).

For comparison purposes, a control mortar reinforced with synthetic fibres (PP) was also tested under the same conditions, as shown in Figure 3.

At 1 day of curing, all wool-reinforced mortars exhibited relatively low compressive strength values, reflecting the early hydration stage of the cementitious matrix. In this initial phase, the incorporation of wool fibres generally led to lower compressive strength compared to the control mortar reinforced with synthetic fibres. This behaviour is commonly reported for fibre-reinforced cementitious materials, particularly when flexible natural fibres are introduced, as they may locally increase porosity and interfere with particle packing before full matrix densification is achieved [9,29].

After 7 days of curing, a substantial increase in compressive strength was observed for all mixtures. Both washed and encapsulated wool mortars showed marked strength gains compared to their 1-day values, indicating that the hydration and consolidation of the cement matrix progressively offset the initial weakening effects associated with fibre incorporation. Among the wool-reinforced mortars, those containing encapsulated fibres generally exhibited higher compressive strength than their washed-wool counterparts at equivalent fibre contents. This trend suggests that fibre encapsulation contributes to a more compatible fibre–matrix interface and limits the formation of weak zones around the fibres, as previously observed in studies involving treated natural fibres in alkaline cementitious environments [10].

At 28 days, compressive strength values continued to increase and reached their maximum for all mixtures. For both washed and encapsulated wool, the highest compressive strength was typically achieved at a fibre content of 1 g per batch. Increasing the fibre dosage to 3 g did not result in further improvements and, in some cases, led to a reduction in compressive strength. This reduction can be attributed to fibre agglomeration and an increase in entrapped air or voids at higher fibre contents, which negatively affect matrix continuity and load transfer under compressive stress [30]. Similar non-linear effects of fibre dosage on compressive strength have been widely reported for natural fibre-reinforced mortars and concretes.

For comparison purposes, a control mortar reinforced with synthetic fibres (PP) was also tested under the same conditions, as shown in Figure 3. As no direct microstructural characterisation (e.g., SEM/porosity) was conducted, the proposed mechanism is discussed as a literature-supported interpretation.

When compared with the control mortar reinforced with synthetic fibres, wool-reinforced mortars generally exhibited lower compressive strength; however, at 28 days, the washed-wool mortar at 1.0 g per batch (W-1) slightly exceeded the PP counterpart at the same dosage (PP-1). This behaviour may be attributed to the fact that, at moderate fibre contents, washed wool can help restrain microcrack development and—due to the moisture-buffering capacity of natural fibres—may promote continued hydration via an internal-curing effect, leading to a locally denser matrix/ITZ [31,32]. By contrast, PP fibres are hydrophobic and can present weaker fibre–matrix interaction and a more porous ITZ, which can limit their contribution to compressive strength at comparable dosages [33,34].

Comparable findings have been reported in the literature, where wool fibres were shown to improve toughness and crack control while maintaining compressive strength within acceptable ranges for non-structural applications [15].

Mechanistically, wool fibres may play a dual role: (i) reinforcement through crack-bridging/constraint, consistent with the flexural response where the optimal washed-wool dosage (W-1) approaches the PP reference; and (ii) an indirect pore-/microstructure-related effect via changes in workability/compaction, entrained air, and moisture-buffering during hardening. The latter is consistent with the fact that W-1 outperforms the PP counterpart in compression at 28 days. Since no direct pore-structure/microstructural measurements were conducted, these mechanisms are discussed as trend-based interpretations.

### 3.2. Physical Properties

This section presents the physical properties of the wool-reinforced mortars at 28 days, focusing on apparent density and water absorption as indicators of the composite’s bulk compactness and moisture-related behaviour.

#### 3.2.1. Apparent Density

**Figure 4:**
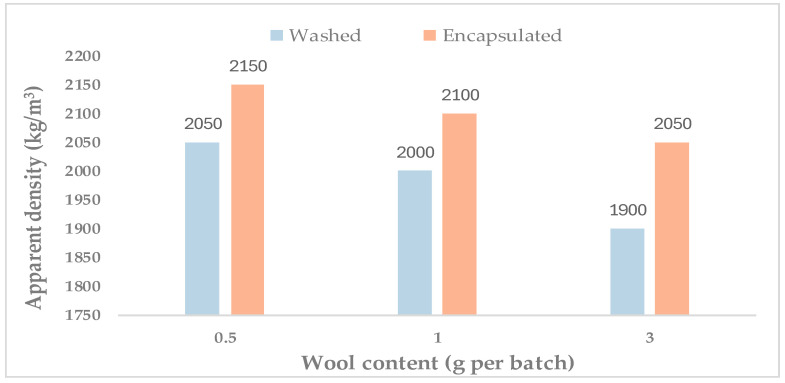
Apparent density at 28 days as a function of wool content (0.5, 1, and 3 g per batch) for mortars incorporating washed and encapsulated wool (n = 1 specimen per mixture; therefore, no error bars are reported).

Figure 4 shows the apparent density at 28 days as a function of wool content (0.5, 1, and 3 g per batch), comparing mixtures containing washed wool and encapsulated wool.

For washed wool (n = 1), density decreased from 2050 kg/m^3^ (0.5 g/batch) to 2000 kg/m^3^ (1 g/batch) and 1900 kg/m^3^ (3 g/batch). The reduction in density with increasing wool dosage is consistent with the fact that wool is a very low-density addition relative to the cement–sand matrix, and its incorporation can lead to a lower bulk density of the hardened composite. Similar behaviour has been reported for rendering mortars reinforced with sheep wool fibres, where the addition of wool reduced dry bulk density due to the low bulk density of the fibres [9]. In addition, natural fibre incorporation in renders has been shown to increase open porosity at 28 days, which would also contribute to lower bulk density through a larger void fraction [35].

At equivalent dosages, the encapsulated-wool mortars exhibited higher apparent density than the washed-wool mortars. This may be attributed to the presence of the cementitious encapsulation layer, which increases the effective mass of the fibre phase and can reduce the extent of density loss relative to washed wool at the same dosage.

This behaviour is consistent with reports on natural-fibre-reinforced cementitious mortars, where fibre addition can increase entrapped air/porosity and reduce bulk density, while fibre treatments that improve fibre–matrix compatibility may limit void formation and lead to slightly higher densities [36,37]. In the present case, washed wool (W) may promote fibre bundling and local voids, whereas cement encapsulation (E) provides a more mineral-like surface that can improve packing/interaction with the paste, contributing to the higher apparent density observed for E.

Finally, the density range measured here (1900–2150 kg/m^3^) remains well above the threshold typically used to classify lightweight mortars (dry hardened density ≤ 1300 kg/m^3^), so these mixtures should be interpreted as conventional-density mortars with modified physical behaviour rather than truly lightweight systems; however, since apparent density was obtained from one specimen per mixture at 28 days, the results are best discussed in terms of trends between fibre treatments and dosages.

#### 3.2.2. Water Absorption

**Figure 5:**
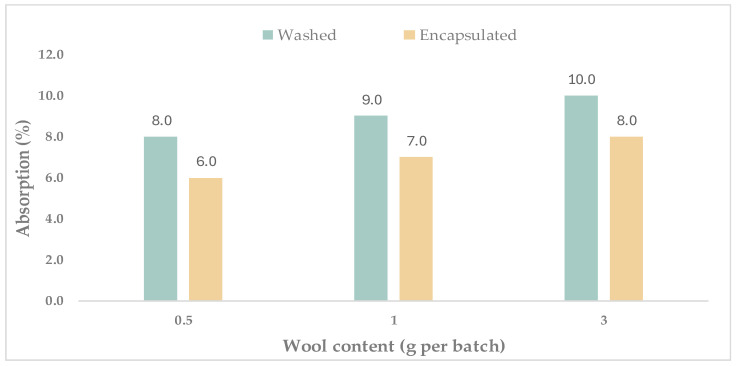
Absorption (%) as a function of wool content (0.5, 1, and 3 g per batch) for specimens containing washed and encapsulated wool; bars indicate the measured absorption values (n = 1 specimen per mixture; therefore, no error bars are reported).

Figure 5 presents the water absorption at 28 days as a function of wool content (0.5, 1, and 3 g per batch) for mortars incorporating washed wool and encapsulated wool; bars indicate the measured absorption values.

The measured water absorption (n = 1) increased with wool content for both fibre conditions. For washed wool, absorption rose from approximately 8% at 0.5 g/batch to ~9% at 1 g/batch and ~10–11% at 3 g/batch. For encapsulated wool, the corresponding values were consistently lower, increasing from ~6% (0.5 g/batch) to ~7% (1 g/batch) and ~8% (3 g/batch).

The overall increase in water uptake with fibre dosage is consistent with the general trend reported for natural-fibre rendering mortars, where fibre incorporation can increase water transport by capillarity and is commonly associated with changes in pore structure and interfacial zones [9,35]. Wool fibres are also strongly hygroscopic, which is widely recognised as a key durability-related aspect of wool–cement composites [10]. At a given dosage, the lower absorption observed for the encapsulated-wool mixtures is plausibly linked to the cementitious coating partially limiting direct fibre wetting and/or reducing preferential pathways along the fibre–matrix interface, although this mechanism would require complementary microstructural evidence to be confirmed.

Finally, absorption by immersion is sensitive to mixture design and testing protocol (notably specimen preconditioning and immersion conditions); therefore, the present values are best interpreted comparatively within the studied series rather than against a single universal benchmark [38].

### 3.3. Thermal Conductivity

This subsection evaluates the influence of sheep wool reinforcement on the heat-transfer behaviour of the hardened mortars at 28 days. Thermal conductivity is reported for the reference mixture and for the highest wool dosage (3 g per batch), considering both washed and cement-encapsulated fibres.

Measurements were performed on plates conditioned to a consistent moisture state (oven-dried at 60 °C for 24 h), and the analysis focuses on the highest wool dosage (W-3 and E-3), which is expected to provide the largest insulation improvement.

Table 7 summarises the measured thermal conductivity (λ) for the control mortar and the two wool-reinforced mortars (W-3 and E-3), obtained in two temperature intervals (25–35 °C and 35–45 °C). Because only one specimen per series was tested (n = 1), no error bars or dispersion metrics are reported.

Compared with the control (λ = 1.85 and 1.82 W·m^−1^·K^−1^), both wool-reinforced mortars exhibited a clear reduction in thermal conductivity.

Washed wool (W-3) yielded the lowest values (1.52 and 1.49 W·m^−1^·K^−1^), corresponding to an observed decrease of ~18% relative to the control across the two temperature intervals, whereas the encapsulated wool mixture (E-3) showed slightly higher values (1.59 and 1.55 W·m^−1^·K^−1^; ~14–15% reduction).

The reduction is consistent with the general expectation that adding sheep wool, whose intrinsic thermal conductivity is very low compared with cementitious matrices, can improve the insulating character of cement-based composites, particularly through the combined effect of a low-conductivity fibrous phase and an associated increase in air voids/porosity [7]. The slightly higher λ of the encapsulated-wool mortar compared with the washed-wool mortar at the same dosage is also coherent with the higher bulk compactness expected when fibres carry an additional cementitious coating (i.e., a denser fibre phase and potentially reduced “insulating” void contribution), which aligns with the density trends observed in Section 3.2.1.

Across the tested temperature intervals, λ decreased marginally for all mixtures (≈0.02–0.04 W·m^−1^·K^−1^). Given the limited dataset (single specimen per series), this small variation is best treated as a descriptive trend rather than a definitive temperature-dependence.

Overall, these results indicate that even at conventional-density levels, wool reinforcement can measurably reduce thermal conductivity, supporting its potential for eco-mortars, where moderate gains in thermal performance are desirable without fully transitioning into lightweight mortar classes [10].

### 3.4. Microstructural Analysis (Microscopy)

This section provides a qualitative assessment of fibre distribution and local matrix features (e.g., visible voids and fibre clustering), using digital microscopy observations to support the trends discussed in Section 3.2 and Section 3.3. In fibre-reinforced cementitious composites, macroscopic behaviour is known to depend strongly on fibre dispersion and local heterogeneity [39].

**Figure 6:**
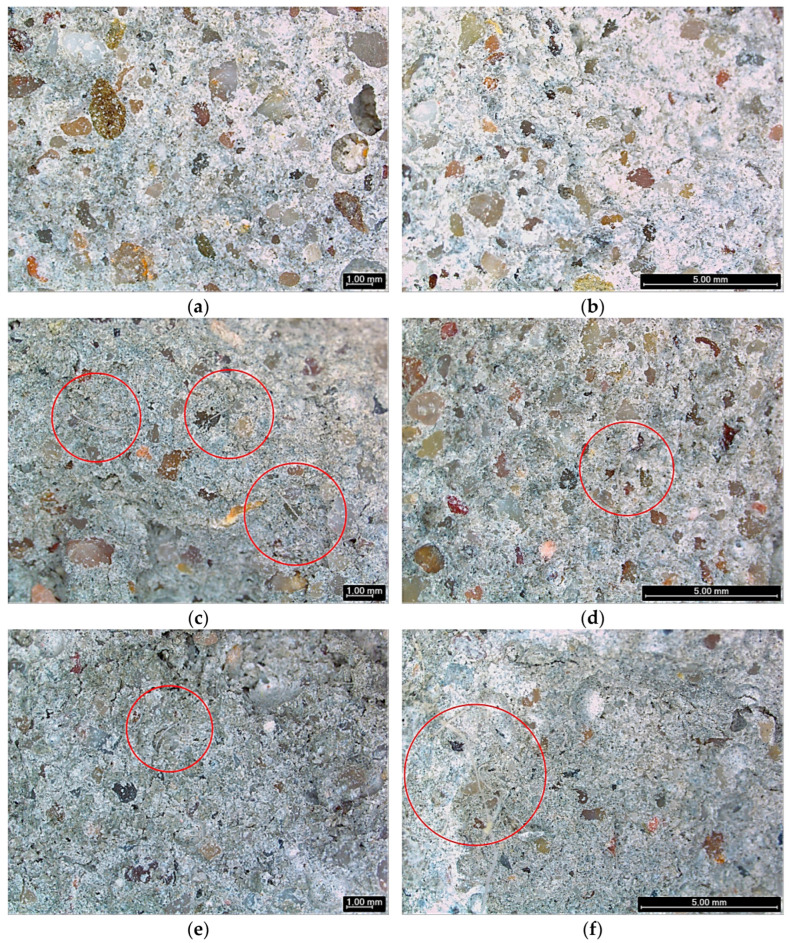
Digital microscopy images (2.4×) of mortars reinforced with washed and cement-encapsulated wool at 0.5, 1, and 3 g per batch: (**a**) washed 0.5; (**b**) encapsulated 0.5; (**c**) washed 1; (**d**) encapsulated 1; (**e**) washed 3; and (**f**) encapsulated 3.

Figure 6 presents representative digital microscopy images of mortars reinforced with washed and cement-encapsulated wool at 0.5, 1, and 3 g per batch, acquired at 2.4× magnification and arranged by dosage and treatment.

At the lowest fibre content (0.5 g per batch), fibres were difficult to identify on the inspected surfaces, and only occasional short fibres could be observed depending on the viewing angle (Figure 6a,b). In this condition, the washed-wool specimen shows a more heterogeneous appearance, with isolated pores visible in the observed area (Figure 6a), whereas the encapsulated-wool specimen does not show comparably large pores in the inspected fragment (Figure 6b).

At 1 g per batch (Figure 6c,d), fibres are more readily observed in the washed-wool mortar (Figure 6c), while fewer fibres are visible in the encapsulated-wool specimen within the examined fragment (Figure 6d). This contrast is consistent with non-uniform fibre distribution at the fragment scale and highlights that local microscopy observations may depend on sampling location (i.e., fibre-rich and fibre-poor zones can coexist within the same mixture), which is a well-known issue in fibre-reinforced cementitious systems [39].

At the highest dosage (3 g per batch), fibre clusters/agglomerations are more frequently observed in both treatments (Figure 6e,f). Qualitatively, washed wool appears as looser and more flexible fibres (Figure 6e), whereas encapsulated wool appears stiffer and more compact, which is consistent with fibres being confined within a cementitious coating (Figure 6f). In wool-reinforced mortars, increased voids/open porosity with wool incorporation has been reported and is commonly associated with interfacial effects and packing disturbances introduced by fibres [9].

More broadly, fibre treatments/surface modifications are widely discussed as strategies to alter fibre–matrix interactions and durability-related behaviour in cement-based fibre composites [40].

Overall, the microscopy observations support two points relevant for interpreting the macroscopic behaviour: (i) increasing wool dosage increases the likelihood of fibre clustering (notably at 3 g per batch), which can locally disturb packing; and (ii) encapsulation modifies fibre morphology/stiffness, plausibly influencing fibre–matrix contact and transport pathways. Since the present observations are qualitative and obtained at low magnification from fragments after destructive testing, higher-resolution techniques (e.g., SEM) and/or quantitative porosity measurements would be required to confirm interfacial features and pore connectivity.

### 3.5. Statistical Analysis

Statistical analysis was applied to the 28-day mechanical responses in order to quantitatively assess the influence of fibre treatment (washed or encapsulated) and fibre content (0.5, 1.0, and 3.0 g per batch) on the ultimate flexural strength (*f_u_*_,*f*_) and ultimate compressive strength (*f_u_*_,*c*_). The analysis focuses on identifying statistically significant main effects and interactions between the studied factors. These statistical results should be interpreted in the context of the available replicates, particularly for flexural strength (N = 12), and are therefore used as supportive evidence rather than definitive proof.

Table 8 shows the results of the analysis of variance for the indicated variables.

For flexural strength (*f_u_*_,*f*_), fibre content exhibited a statistically significant effect (*p* = 0.023), whereas fibre treatment did not show a significant influence (*p* = 0.325). The interaction between treatment and fibre content was also not statistically significant (*p* = 0.128), indicating that the effect of fibre content on *f_u_*_,*f*_ is largely independent of the wool treatment condition within the studied range.

In contrast, the compressive strength (*f_u_*_,*c*_) showed statistically significant main effects of both fibre treatment (*p* = 0.009) and fibre content (*p* < 0.001), together with a highly significant treatment × content interaction (*p* < 0.001). This result indicates that the influence of fibre content on compressive strength strongly depends on whether the wool fibres are washed or encapsulated.

The goodness-of-fit of the general linear models fitted to the 28-day mechanical responses is summarised in Table 9.

For flexural strength, the model exhibits a moderate explanatory capability, as indicated by an R^2^ value of 78.75% and a standard error of the regression S = 0.64 MPa.

In contrast, the model fitted to compressive strength shows a markedly stronger fit, with an R^2^ value of 90.85% and a higher but still acceptable regression error (S = 3.12 MPa), reflecting the larger absolute magnitude of compressive strength values.

The fitted relationships are expressed by the effect-coded regression Equations (2) and (3) for *f_u_*_,*f*_ and *f_u_*_,*c*_, respectively, which provide a quantitative representation of the influence of fibre treatment, fibre content, and their interaction on the 28-day mechanical behaviour:

(2) $$f_{u,f}=8.567+0.199\cdot I_{E}-0.199\cdot I_{W}+0.333\cdot I_{0.5}+0.673\cdot I_{1.0}-1.005\cdot I_{3.0}\;+0.481\cdot I_{E}\cdot I_{0.5}-0.604\cdot I_{E}\cdot I_{1.0}+\;0.123\cdot I_{E}\cdot I_{3.0}-0.481\;\cdot I_{W}\cdot I_{0.5}+0.604\cdot I_{W}\cdot I_{1.0}-0.123\cdot I_{W}\cdot I_{3.0}$$

(3) $$f_{u,c}=44.876+1.865\cdot I_{E}-1.865\cdot I_{W}+3.610\cdot I_{0.5}+4.895\cdot I_{1.0}-8.505\cdot I_{3.0}\;+4.688\cdot I_{E}\cdot I_{0.5}-8.064\cdot I_{E}\cdot I_{1.0}+3.376\cdot I_{E}\cdot I_{3.0}-4.688\;\cdot I_{W}\cdot I_{0.5}+8.064\cdot I_{W}\cdot I_{1.0}-3.376\cdot I_{W}\cdot I_{3.0}$$

It must be noted that $I_{E}$ and $I_{W}$ denote the effect-coded indicator variables associated with the fibre treatment (encapsulated and washed wool, respectively), while $I_{0.5}$, $I_{1.0}$, and $I_{3.0}$ denote the effect-coded indicator variables corresponding to fibre contents of 0.5, 1.0, and 3.0 g per batch. Effect coding was adopted for categorical factors, such that $I_{E}+I_{W}=0$ and $I_{0.5}+I_{1.0}+I_{3.0}=0$. Interaction terms are defined as products of the corresponding indicator variables. Coefficients, therefore, represent deviations from the overall mean response.

**Figure 7:**
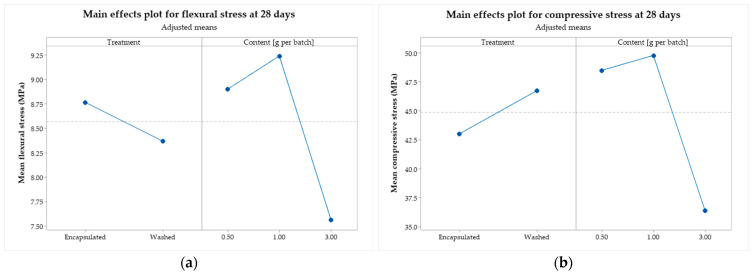
Main effects plots showing the influence of fibre treatment and fibre content on the responses obtained from mechanical tests: (**a**) ultimate flexural strength, and (**b**) ultimate compressive strength.

Finally, the main effects plots are provided in the accompanying Figure 7. As can be seen, the plots highlight a pronounced effect of fibre content on the flexural strength, with the highest adjusted mean observed at 1.0 g per batch and a marked reduction at 3.0 g per batch, whereas the difference between washed and encapsulated wool remains comparatively small. On the other hand, the main effects plots reveal a stronger influence of both factors on the compressive strength, with substantial variations associated with changes in fibre content and a clear difference between treatments. These trends are consistent with the statistically significant main effects identified by the ANOVA and visually support the interaction-driven behaviour observed for compressive strength.

## 4. Conclusions

This study demonstrates that sheep wool fibres can be effectively incorporated into cement-based mortars as a sustainable reinforcement, providing mechanically stable materials while offering additional functional and environmental benefits:

- At 28 days, the washed wool mixture with 1.0 g per batch (W-1) delivered the best flexural performance among the wool-reinforced mortars, reaching 9.65 ± 0.50 MPa and closely matching the PP-reinforced reference under bending.
- In compression, the washed wool mixture at 1.0 g per batch (W-1) also provided the strongest response, achieving 59.70 ± 1.05 MPa at 28 days and outperforming the PP-reinforced reference. This result highlights that, at an optimal dosage, washed wool fibres can deliver not only competitive but even superior compressive performance compared with the synthetic benchmark.
- ANOVA confirmed that fibre dosage dominates the mechanical response, while treatment plays a secondary role in flexure but becomes more influential in compression through its interaction with fibre–matrix bonding.
- Beyond mechanical performance, wool incorporation led to lower thermal conductivity (up to ~18% observed reduction; n = 1 per series) together with reductions in apparent density, supporting the development of multifunctional mortars where mechanical performance is complemented by improved thermal behaviour.
- Mechanical performance was evaluated up to 28 days only; long-term alkaline durability of wool/keratin and its implications for strength and crack-bridging will be addressed in future work through extended-age testing and accelerated ageing.

The findings highlight the potential of sheep wool fibres as a competitive and eco-efficient reinforcement in multifunctional cementitious systems, offering a practical route to more sustainable construction and to the circular-economy valorisation of agricultural by-products, particularly where multifunctionality is prioritised over purely structural performance.

## Figures and Tables

**Table 1:** OPC main components and density. Reproduced from Ref. [19] (CC BY 4.0).

Main Components XRF (%)	CaO	SiO_2_	SO_3_	Al_2_O_3_	Fe_2_O_3_	MgO	K_2_O	Na_2_O	TiO_2_	Density (g/cm^3^) (UNE-EN 196-6) [20]
CEM I 42.5R	66.22	17.95	5.44	4.25	2.89	1.36	1.12	0.39	0.19	3.11

**Table 2:** Morphological and visual characterisation of Segureña sheep wool fibres, including colour, type, and quantity of residues, along with the fibre diameter measurements obtained by optical microscopy. Adapted from Ref. [21] (CC BY 4.0); edited for clarity.

Figure	Colour ^1^	Residue Type ^1^	Residue Quantity ^1^	Max. Fibre Diam. (μm)	Min. Fibre Diam. (μm)	Mean Fibre Diam. (μm)
Figure 1a	Light white	Straw	Low	70	20	45
Figure 1b	White/yellow	Soil/straw	Low	60	30	45

^1^ Values based on subjective visual assessment.

**Table 3:** Material dosages for the preparation of mortar batches.

Mixture ID	Fibre Treatment/Type	Fibre Mass (g per Batch)	Cement (g)	Sand (g)	Water (g)	Fibre Content (Bfoc, wt.%)
Control	Unreinforced	0	450	1350	225	0.00
PP-0.5	PP microfibre	0.5	450	1350	225	0.11
PP-1	PP microfibre	1	450	1350	225	0.22
PP-3	PP microfibre	3	450	1350	225	0.67
W-0.5	Washed wool	0.5	450	1350	225	0.11
W-1	Washed wool	1	450	1350	225	0.22
W-3	Washed wool	3	450	1350	225	0.67
E-0.5	Encapsulated wool	0.5	450	1350	225	0.11
E-1	Encapsulated wool	1	450	1350	225	0.22
E-3	Encapsulated wool	3	450	1350	225	0.67

**Table 4:** Summary of the experimental programme: test types, mixtures, curing ages, specimen geometry, and number of specimens.

Test	Mixtures Included	Curing Ages (Days)	Specimen Type and Dimensions	Replicates
Consistency (flow table)	All mixtures	Fresh state	Fresh mortar	1 test per batch
Flexural strength	All mixtures	1, 7, 28	Prism (40 × 40 × 160 mm)	1 at 1 day; 2 at 7 and 28 days
Compressive strength	All mixtures	1, 7, 28	Half-prism	2 at 1 day, 4 at 7, and 28 days
Water absorption	All mixtures	28	Prism (40 × 40 × 160 mm)	1 per mixture
Apparent density	All mixtures	28	Prism (40 × 40 × 160 mm)	1 per mixture
Thermal conductivity	Control; W-3, E-3	28	Plate (300 × 300 × 30 mm)	1 specimen per series
Microstructural analysis	All mixtures	28	Fragment from cured prism	1 observed per mixture

**Table 5:** Flexural strength of wool-reinforced mortars at different curing ages.

Specimen	Day 1 (MPa)	Day 7 (MPa)	Day 28 (MPa)
W-0.5	1.12	8.24 ± 2.03	8.22 ± 0.59
W-1	3.27	7.90 ± 0.01	9.65 ± 0.50
W-3	1.97	5.47 ± 0.38	7.24 ± 0.04
E-0.5	5.99	7.59 ± 0.86	9.58 ± 0.58
E-1	5.87	6.53 ± 0.57	8.84 ± 1.24
E-3	2.88	6.34 ± 0.23	7.89 ± 0.11

**Table 6:** Compressive strength of wool-reinforced mortars at different curing ages.

Specimen	Day 1 (MPa)	Day 7 (MPa)	Day 28 (MPa)
W-0.5	3.98 ± 0.05	29.53 ± 0.74	45.66 ± 1.53
W-1	10.66 ± 0.83	41.64 ± 4.03	59.70 ± 1.05
W-3	4.81 ± 0.02	20.73 ± 2.33	34.86 ± 0.93
E-0.5	4.93 ± 0.21	32.03 ± 3.91	51.31 ± 2.49
E-1	3.69 ± 2.04	26.62 ± 0.87	39.84 ± 5.78
E-3	7.87 ± 0.28	28.72 ± 1.69	37.88 ± 3.84

**Table 7:** Thermal conductivity (λ) of the reference mortar and mortars reinforced with 3 g/batch washed (W-3) and encapsulated wool (E-3), measured at two temperature intervals.

Mixture	Wool Treatment	Wool Content [g per Batch]	Thermal Conductivity (W·m^−1^·K^−1^)	
			25–35 °C	35–45 °C
Control (unreinforced)	-	0	1.85	1.82
W-3	Washed	3	1.52	1.49
E-3	Encapsulated	3	1.59	1.55

**Table 8:** Analysis of variance (ANOVA) results for the response variables: *f_u_*_,*f*_ and *f_u_*_,*c*_.

Test	Variable	Source	DF	Adj SS	Adj MS	F-Value	*p*-Value
Flexural	Flexural strength (*f_u_*_,*f*_)	Treatment	1	0.4760	0.4760	1.15	0.325
		Fibre Content [g per batch]	2	6.2914	3.1457	7.59	0.023
		Treatment × Fibre content	2	2.4457	1.2229	2.95	0.128
		Error	6	2.4854	0.4142		
		Total	11	11.6984			
Compressive	Compressive strength (*f_u_*_,*c*_)	Treatment	1	83.44	83.440	8.55	0.009
		Fibre Content (g per batch)	2	874.63	437.313	44.80	<0.001
		Treatment × Fibre content	2	787.26	393.630	40.32	<0.001
		Error	18	175.72	9.762		
		Total	23	1921.05			

**Table 9:** Summary of the regression models for the response variables: *f_u_*_,*f*_ and *f_u_*_,*c*_.

Variable	S	R^2^ [%]
Ultimate Flexural Strength, *f_u_*_,*f*_ [MPa]	0.643603	78.75
Ultimate Compressive Strength, *f_u_*_,*c*_ [MPa]	3.12450	90.85

## Data Availability

The original contributions presented in this study are included in the article. Further inquiries can be directed to the corresponding author.
